# A Case of Systemic Lupus Erythematosus/Antineutrophil Cytoplasmic Antibody-Associated Vasculitis Overlap Syndrome with Dissociated Pathological and Immunological Findings

**DOI:** 10.1155/2020/5698708

**Published:** 2020-05-15

**Authors:** Kazuhiko Kato, Tetsuya Kawamura, Risa Terashima, Yukiko Tsuchiya, Yasuhito Takahashi, Kenji Kasai, Takashi Yokoo

**Affiliations:** ^1^Department of Internal Medicine, Fuji City General Hospital, Takashima-cho 50, Fuji-shi, Shizuoka, Japan; ^2^Division of Nephrology and Hypertension, Department of Internal Medicine, The Jikei University School of Medicine, Tokyo, Japan

## Abstract

Systemic lupus erythematosus/antineutrophil cytoplasmic antibody-associated vasculitis overlap syndrome (SLE/AAV OS) describes a pathological condition that presents with overlapping features of two diseases. There have been few reports of SLE/AAV OS and none from Japan. We present the case of a 59-year-old woman admitted with chief complaints of fever and decreased renal function. SLE was suspected due to the identification of four items from the diagnostic criteria of the American College of Rheumatology, including positivity for anti-ds-DNA and antinuclear antibodies. However, pathological findings from the kidney biopsy suggested pauci-immune crescentic glomerulonephritis. She was also diagnosed with AAV according to the Chapel Hill Consensus Conference (CHCC) 2012 definitions and the classification algorithm of AAV. SLE/AAV OS was suspected, we started immunosuppressant therapy, and subsequently her renal function improved. In previous reports, initial immunological and pathological findings generally concur. In cases where clinical and pathological features appear to conflict, as in the present case, a treatment strategy decision should be based on pathological and immunological findings to improve the prognosis of OS.

## 1. Introduction

Systemic lupus erythematosus/antineutrophil cytoplasmic antibody-associated vasculitis overlap syndrome (SLE/AAV OS) is a disease entity proposed by Nasr et al. in 2008 [1]. This condition presents with overlapping features of two diseases in terms of physical, laboratory, and pathological findings. Previously reported cases [1–5] have described women with a mean age of 40 years who presented with symptoms characteristic of two diseases: arthritis, including cytopenia, rhinitis, pulmonary hemorrhage, and interstitial pneumonia, and kidney changes, including hematuria and proteinuria, often exhibiting rapid progression to glomerulonephritis. SLE/AAV OS exhibits features of both SLE and AAV but, in many cases, the features of one of the two diseases are more evident; in addition, the pathological and immunological findings of the more evident disease, including various autoantibodies, are predominant. No treatment has been established for SLE/AAV OS, and disease management decisions must be based on the more prominent features of either SLE or AAV. In past reports [1–5], the initial immunological and pathological findings often agreed; however, they were inconsistent in the present case.

## 2. Case Presentation

A 59-year-old woman with no family history of kidney disease was receiving treatment for hypertension and dyslipidemia. The patient was taking oral olmesartan medoxomil (20 mg once daily), oral amlodipinebesylate (10 mg once daily), and oral rosuvastatin calcium (2.5 mg once daily). She had been experiencing symptoms of a common cold for approximately one month and developed a night fever 14 days before admission. Oral antipyretics and similar drugs did not improve her symptoms. Her serum creatinine level reached 2.4 mg/dL 14 days before admission and increased to 3.6 mg/dL on the day of admission, which was the day when she was referred to our hospital for an investigation of her worsening renal function. Findings on her arrival were as follows: body height, 160 cm; body weight, 60 kg; blood pressure, 138/80 mmHg; pulse, 80 beats/min; body temperature, 37.3°C; no anemia in the palpebral conjunctiva; breathing sounds were clear/no secondary noises; no heart murmurs; no edema in the legs; no joint pain or eruption; and no oral enanthem.

Blood test results on admission were as follows: white blood cell count, 12,900 cells/*μ*L; lymphocytes, 800 cells/*μ*L; monocytes, 11,000 cells/*μ*L; neutrocytes, 10,800 cells/*μ*L; eosinophils, 100 cells/*μ*L; basophils, 0 cells/*μ*L; red blood cell count, 3.57 × 10⁶ cells/*μ*L; hemoglobin, 10.9 g/dL; hematocrit, 31.7%; platelets, 350 × 10³ cells/*μ*L; aspartate transaminase, 62 IU/L; alanine transaminase, 119 IU/L; lactate dehydrogenase, 289 IU/L; total protein, 6.3 g/dL; albumin, 2.3 g/dL; urea nitrogen, 35 mg/dL; creatinine, 3.73 mg/dL; sodium, 136 mmol/L; potassium, 3.8 mmol/L; calcium, 8.0 mg/dL; phosphate, 4.8 mg/dL; C-reactive protein, 14.25 mg/dL; immunoglobulin G, 1,169 mg/dL; immunoglobulin A, 413 mg/dL; immunoglobulin M, 98 mg/dL; complement component, 3 132 mg/dL; complement component, 4 30.3 mg/dL; and 50% hemolytic complement, 98.8 U/mL. Furthermore, serine proteinase 3 antineutrophil cytoplasmic antibody, <0.51 IU/mL; myeloperoxidase antineutrophil cytoplasmic antibody (MPO-ANCA), 8.2 IU/mL; anti-double-strand (ds)-DNA antibody IgG, 54.9 IU/mL; anti-Smith antibody, negative; anticardiolipin immunoglobulin, 1 U/mL; and lupus anticoagulant, negative. Antinuclear antibody homogeneous pattern 160-fold and nucleolar pattern 80-fold were identified.

Urine testing revealed proteinuria (2081 mg/day) and hematuria (red blood cells ≥ 100/high-power field (poikilocytes 0–10%)). Plain thoracoabdominal computed tomography indicated reticular shadows in both inferior lobes. Renal size was preserved.

**Figure 1:**
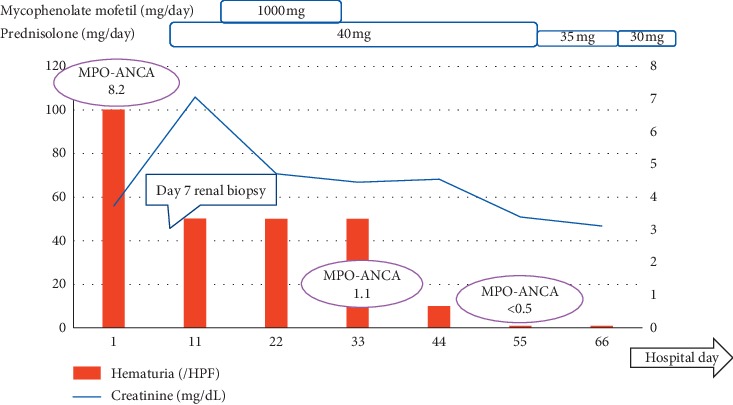
Disease course after admission. The patient was suspected of SLE/AAV OS with serological features of SLE, and the treatment was changed to PSL monotherapy on day 8. Thereafter, the dosage of PSL was gradually decreased, and serum creatinine levels improved from a maximum of 7.06 mg/dL to 3.39 mg/dL on day 56.

We suspected SLE because four items from the diagnostic criteria of the American College of Rheumatology (ACR) were satisfied: presence of antinuclear antibodies, positivity for anti-ds-DNA antibody, serum lymphocytopenia, and urine protein of 2.0 g/day. Treatment with mycophenolate mofetil (MMF) (1000 mg/day, orally) and prednisolone (PSL) (40 mg/day, orally) was started for SLE/lupus nephritis. On the 32^nd^ hospital day, results of the renal biopsy that was taken on the 7th hospital day were confirmed. The disease course after admission is shown in Figure 1.

**Figure 2:**
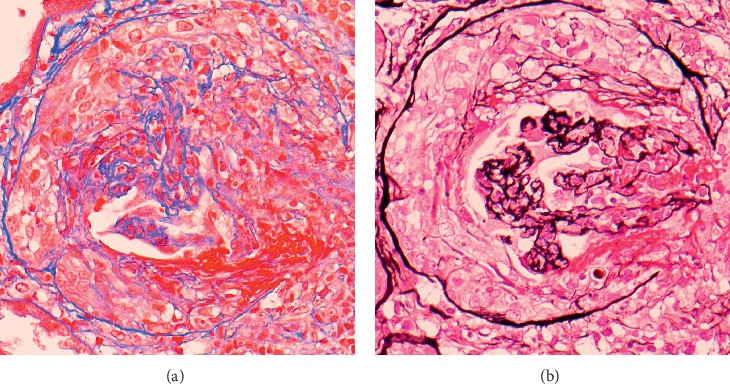
Global sclerosis was observed in two of a total 17 glomeruli. Cellular crescents and fibrinoid necrosis with rupture of Bowman capsule were observed in 14 of the remaining 15 glomeruli. (a) Masson's trichrome stain and (b) PAM stain showed cellular crescents and fibrinoid necrosis, with Bowman's capsule rupture.

Global sclerosis was observed in two of a total 17 glomeruli. Cellular crescents and fibrinoid necrosis with rupture of Bowman capsule were observed in 14 of the remaining 15 glomeruli (Figure 2).

**Figure 3:**
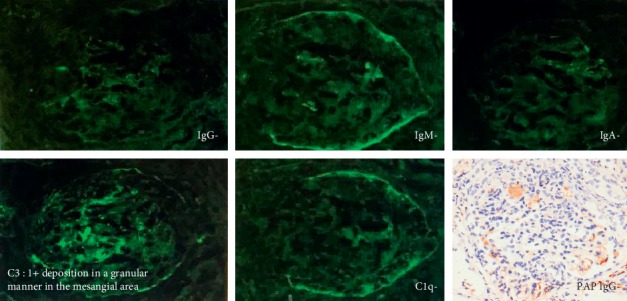
Immunofluorescence showed pauci-immune type crescentic glomerulonephritis (1+ deposition in a granular manner in the mesangial area of C3 only). Peroxidase antiperoxidase (PAP) method showed no deposition of IgG in the glomerular tuft free of crescent.

The degree of tubulointerstitial injury was 85%. Immunofluorescence showed pauci-immune type crescentic glomerulonephritis (1+ deposition in a granular manner in the mesangial area of C3 only) (Figure 3).

She was diagnosed as AAV according to the Chapel Hill Consensus Conference (CHCC) 2012 definitions [6] and the classification algorithm of AAV by Watts et al. [7]. She was suspected of SLE/AAV OS with serological features of SLE, and the treatment was changed to PSL monotherapy on day 8. Thereafter, the dosage of PSL was gradually decreased, and serum creatinine levels improved from a maximum of 7.06 mg/dL to 3.39 mg/dL on day 56.

## 3. Discussion

SLE is an autoimmune disease caused by the deposition of autoantibodies, including antinuclear antibodies [8]. Various clinical symptoms are associated with SLE, and 30–60% of patients present with lupus nephritis (LN) [9, 10].

The diagnostic criteria of the ACR are widely used for the diagnosis of SLE. The application of these criteria for the diagnosis of Japanese patients has been reported to have a sensitivity of 97% and specificity of 89% [11]. Double-stranded-DNA antibodies are specific to SLE, and the specificity of this approach is reportedly 97.4% [12].

LN is characterized by immune-complex deposition in the glomeruli [13]; specifically, pathological findings show deposition in the glomerular basement membrane [14]. Thus, the renal biopsy and assessment of pathological findings from a renal lesion are useful for diagnosing LN/SLE.

The autoimmune disease AAV targets small- and medium-sized blood vessels and is characterized by ANCA-positive laboratory results [15]. This disease has three possible patterns: granulomatosis with polyangiitis, microscopic polyangiitis, or eosinophilic granulomatosis with polyangiitis [16]. Renal pathological findings include the absence of immune-complex deposition in the glomeruli, and the condition is characterized by pauci-immune necrotizing crescentic glomerulonephritis [17]. Our patient was also diagnosed as AAV according to the Chapel Hill Consensus Conference (CHCC) 2012 definitions [6] and the classification algorithm of AAV by Watts et al. [7].

A positive result for ANCA is not representative of a classic presentation of SLE; however, approximately 20% of cases are reportedly positive for ANCA [18–20]. It has been reported that antinuclear antibodies produce p-ANCA-like fluorescence during indirect immunofluorescence screening [21]; results of enzyme-linked immunosorbent assay (ELISA) are also thought to be affected by a cross-reaction with coexisting autoantibodies. Therefore, a positive ANCA status is of little clinical significance for SLE, and it is thought that overlap of the pathology of AAV with ANCA-positive status in SLE patients is very rare [22]. Crescentic nephritis is a feature of ANCA vasculitis, which is frequently observed in severe LN [2]. In particular, LN patients with focal and segmental glomerular inflammation and necrosis that lack immune deposits, similar to AAV, have been described [23]. From reports of patients with symptoms of SLE, positive ANCA status, and AAV-like renal pathological findings, it was concluded that a condition exists in which the two diseases overlap. In 2008, SLE/AAV overlap syndrome was proposed as a disease concept.

In the present case, we identified four items from the ACR diagnostic criteria, including strong positivity for ds-DNA and presence of antinuclear antibodies. The patient was suspected of SLE and was treated with PSL and MMF. Results from the renal biopsy confirmed pauci-immune crescentic glomerulonephritis. Electron microscopy had not detected immune-complex deposition, and the formation of crescents was observed. Immunostaining was positive for C3 only, but some reports of immunofluorescence and electron microscopy in the context of AAV have shown complement deposition in the glomeruli [24]; this is believed to be consistent with the pauci-immune type. The patient was diagnosed as AAV according to the Chapel Hill Consensus Conference (CHCC) 2012 definitions [6] and the classification algorithm of AAV by Watts et al. [7]. Given the above criteria, the patient was diagnosed with AAV.

We could not definitively diagnose SLE because this case did not meet four items from the diagnostic criteria of ACR. However, we suspected SLE/AAV OS with features of SLE. The patient's treatment was changed to PSL monotherapy.

There are only a few case reports [1–5] describing SLE/AAV OS in detail, and no cases of SLE/AAV OS have been reported in Japan. To date, 10 cases of SLE/AAV OS have been reported by Nasr et al. in 2008 [1], four cases by Hervier et al. in 2012 [2], and eight cases by Jarrot et al. in 2016 [3]. Recently, there has been one case reported in 2018 [4] and one in 2019 [5]. Of note, most of these cases had glomerular deposition of immunoglobulin, and only three cases showed pauci-immune glomerulonephritis similar to the present case. In this regard, the present case was rare among patients with SLE/AAV OS.

Many of these cases involved women with a mean age of 40 years. SLE/AAV OS presents with symptoms characteristic of the two diseases; Table 1 lists the serological and pathological features of the 21 reported cases described above (case 4 of [2] was excluded because no renal biopsy was reported). SLE/AAV OS has characteristics of both diseases, but it does not necessarily meet all the diagnostic criteria for both diseases. Likewise, the features of either SLE or AAV are generally more evident, and the pathological and immunological findings, including various autoantibodies, often agree. While many cases are positive for both antinuclear antibodies and ANCA, few are positive for both ANCA and ds-DNA, as was observed in the present case. Cases 5 and 8 of [3] were strongly positive for both ds-DNA antibodies and ANCA, but the renal pathological findings were LN, and the SLE elements appeared to be stronger than those of the present case.

Serological examination of our patient revealed clinical features classified as SLE, and the patient was strongly positive for ds-DNA. Elevation of ANCA was mild, and the pathological conditions were assumed to be SLE. However, renal biopsy findings showed pauci-immune crescentic glomerulonephritis, which revealed inconsistency between the clinical and pathological features. After PSL monotherapy, renal function improved, which brought serum creatinine levels to 3 mg/dL at discharge.

Many past studies [1–5] have reported treatment plans for SLE/AAV OS based on corticosteroids and cyclophosphamide, but outcomes have varied from significant improvement in general condition/renal function to death. Although there are established treatment protocols for SLE and AAV, no treatment method has been established for SLE/AAV OS. When immunosuppressants such as PSL are used, the therapeutic approach must be based on an evaluation of whether elements of SLE or AAV are more prominent. In past reports [1–5], serological and pathological features often align, but there have been some cases published where findings dissociate, as observed in the present case. In this context, it is difficult to assess patients by serological examination alone. The presentation of OS often reflects the pathology of microscopic polyangiitis, and many cases are characterized by renal involvement in which pathological diagnosis is only possible with a kidney biopsy. With SLE/AAV OS, performing a renal biopsy and planning treatment strategies based on pathological findings may improve the outcomes of SLE/AAV OS.

Though a rare disease, SLE/AAV OS causes damage to all organs of the body, including the kidneys. Further investigation is needed to elucidate the underlying pathological mechanisms and to establish methods of treatment that may mitigate the potentially serious consequences of this disease. Here, we presented a rare case where renal biopsy enabled the diagnosis of SLE/AAV OS. Renal biopsy should be considered an important tool for diagnosing SLE/AAV OS and planning treatment strategies.

## Figures and Tables

**Table 1:** Serological and pathological features of the 21 reported cases described above (case 4 of [2] was excluded because no renal biopsy was reported).

	Age	Sex	Hematuria	Proteinuria (g/day)	Serum creatinine (mg/dL)	ANA	Anti-dsDNA (UL/mL)	Low C3/C4	ANCA specificity	Renal biopsy	Positive immunenoreactanats	Diagnosis	Treatment
Case 1 of [1]	22	Female	Yes	1.5	2.7	Positive (1:1286)	Negative	Not determined	MPO-ANCA		IgG 3+ IgM 2+ IgA+ C3 3+ C1q 2+	LN IV-S (A/C)/ANCA GN	Intavenous CY, PSL, MMF
Case 2 of [1]	37	Female	Yes	5.9	10	Positive (1:80)	Positive	Not determined	MPO-ANCA		IgG 3+ C3 3+ C1q 1+	LN V/ANCA GN	IntravenousCY.PSL, azathioprine
Case 3 of [1]	62	Female	Yes	2	4.7	Positive (1:848)	Positive	Not determined	Negative		IgG+ IgM+ C3 2+	LN III (A/C)/ANCA GN	IntravenousCY, PSL
Case 4 of [1]	80	Female	Yes	4	4.5	Positive (1:2560)	Negative	Not determined	Negative		IgG+ IgM+	LN II/ANCA GN	Hydralazine, oral CY, PSL, PLX
Case 5 of [1]	19	Male	Yes	4	9.6	Positive (1:160)	Positive	Not determined	Negative		IgG 3+ IgA+ C3 2+ C1q 2+	LN IV G (A)/ANCA vasculitis	M-PRED, intavenous CY, oral CY, PSL
Case 6 of [1]	50	Female	Yes	3.2	4.4	Positive (1:160)	Negative	Not determined	MPO-ANCA		IgG3+ IgA+ C3 2+ C1q 2+	LN III (A)/ANCA GN	M-PRED, intravenous CY, PSL
Case 7 of [1]	55	Male	Yes	3	21	Positive (1:160)	Positive	Not determined	Negative		IgG 2+ C3 2+	LN V /ANCA GN	PSL,intravenous CY
Case 8 of [1]	37	Female	Yes	6.4	1.1	Positive (1:40)	Negative	Not determined	MPO-ANCA		IgG 3+ C3 2+	LN V /ANCA GN	IntravenousCY, PSL
Case 9 of [1]	44	Female	Yes	1.7	8.8	Positive (1:320)	Positive	Not determined	Negative		IgG 2+ IgM 1+ IgA 1+ C3 2+	LN III (A/C)/ANCA GN	IntravenousCY, PSL,MMF, HC
Case 10 of [1]	78	Female	Yes	3	4.5	Positive (1:640)	Negative	Not determined	MPO-ANCA,PR-3 ANCA		IgG 2+ C3 2+	LN III(A/C)/ANCA GN	Hydralazine, M-PRED, OSL, oral CY
[4]	40	Female	Not determined	Not determined	8.7	Positive (1:640)	Negative	Not determined	MPO-ANCA		Not determined	Pauci-immune GN	PSL, rituximab, HC, CY
[5]	48	Female	Yes	0.6	Not determined	Positive (1:40)	Negative	No/no	PR3-ANCA		IgG+ C3+	LN V	PSL, intravenous CY
Case 1 of [3]	23	Female	Yes	0.8	1.02	Positive (1:800)	23	No/yes	Negative		C3+	Pauci-immune GN	Not determined
Case 2 of [3]	43	Female	Yes	3.2	7.11	Positive (1:1280)	Negative	No/no	MPO-ANCA		IgG+ IgM+ C3+	Pauci-immune GN	Not determined
Case 3 of [3]	57	Female	Yes	3.6	5.63	Positive (1:1280)	Negative	No/no	MPO-ANCA		Pauci-immune	Pauci-immune GN	Not determined
Case 4 of [3]	29	Female	Yes	0.3	1.01	Positive (1:640)	20	No/no	MPO-ANCA		IgG 3+ IgM 2+ C1q 2+	LN II-G	Not determined
Case 5 of [3]	41	Female	Yes	4	1.53	Positive (1:2560)	50	Yes/yes	MPO-ANCA		IgG 2+ IgA 2+ C3 3+ C1q 2+	LN IV-G	Not determined
Case 6 of [3]	53	Female	Yes	6	1.62	Positive (1:600)	Negative	Yes/yes	MPO-ANCA		Negative	Pauci-immune GN	Not determined
Case 7 of [3]	27	Female	Yes	0.8	3.93	Positive (1:200)	22	No/yes	MPO-ANCA		IgG+ C3+	Pauci-immune GN	Not determined
Case 8 of [3]	74	Female	Yes	1	3.31	Positive (1:5240)	50	No/no	MPO-ANCA		IgG 2+ C3 2+	LN IV-G	Not determined
Case 1 of [4]	74	Female	Yes	0.6	4.1	Positive (1:5120)	Positive	No/yes	MPO-ANCA		IgG+ IgM+ C3+	Not determined	Not determined
Case 2 of [4]	35	Female	Yes	＞6	3.4	Positive (1:620)	Positive	No/no	MPO-ANCA		IgG+	Not determined	PSL, CY
Case 3 of [4]	21	Female	Yes	2.5	3.8	Positive (1:2560)	Positive	No/no	MPO-ANCA		IgG+ IgM+ C3+ C1q+	Not determined	PSL, oral CY, intravenous CY

ANA: antinuclear antibody, ANCA: antineutrophil cytoplasmic antibodies, LN: lupus nephritis, GN: glomerulonephritis, CY: cyclophosphamide, MMF: mycophenolate mofetil, PSL: prednisolone, M-PRED: pulse methylprednisolone, PLX: plasmapheresis, and HC: hydroxychloroquine.

## References

[1] Nasr S. H., D’Agati V. D., Park H.-R. (2008). Necrotizing and crescentic lupus nephritis with antineutrophil cytoplasmic antibody seropositivity. *Clinical Journal of the American Society of Nephrology*.

[2] Hervier B., Hamidou M., Haroche J., Durant C., Mathian A., Amoura Z. (2012). Systemic lupus erythematosus associated with ANCA-associated vasculitis: an overlapping syndrome?. *Rheumatology International*.

[3] Jarrot P. A., Chiche L., Hervier B. (2016). Systemic lupus erythematosus and antineutrophil cytoplasmic antibody-associated vasculitis overlap syndrome in patients with biopsy-proven glomerulonephritis. *Medicine (Baltimore)*.

[4] Curtiss P., Liebman T., Khorolsky C., Brinster N., Beasley J., Lo Sicco K. (2018). Systemic lupus erythematosus and antineutrophil cytoplasmic antibody-associated vasculitis: an emerging overlap syndrome with cutaneous manifestations. *JAAD Case Reports*.

[5] Itikyala S., Pattanaik D., Raza S. (2019). Systemic lupus erythematosus (SLE) and antineutrophil cytoplasmic antibody-associated vasculitis (AAV) overlap syndrome: case report and review of the literature. *Case Reports in Rheumatology*.

[6] Sunderkötter C., Lamprecht P., Mahr A., Metze D., Zelger B. (2018). Nomenklatur der kutanen Vaskulitiden - deutschsprachige Definitionen des Dermatologischen Anhanges zur Chapel Hill Consensus Conference. *Journal der Deutschen Dermatologischen Gesellschaft*.

[7] Watts R., Lane S., Hanslik T. (2007). Development and validation of a consensus methodology for the classification of the ANCA-associated vasculitides and polyarteritis nodosa for epidemiological studies. *Annals of the Rheumatic Diseases*.

[8] Ippolito A., Wallace D., Gladman D. (2011). Autoantibodies in systemic lupus erythematosus: comparison of historical and current assessment of seropositivity. *Lupus*.

[9] Cervera R., Khamashta M. A., Font J. (2003). Morbidity and mortality in systemic lupus erythematosus during a 10-year period. *Medicine*.

[10] Maroz N., Segal M. S. (2013). Lupus nephritis and end-stage kidney disease. *The American Journal of the Medical Sciences*.

[11] Yokohari R., Tsunematsu T. (1985). Application, to Japanese patients, of the 1982 American Rheumatism Association revised criteria for the classification of systemic lupus erythematosus. *Arthritis & Rheumatism*.

[12] Kavanaugh A. F., Solomon D. H. (2002). Guidelines for immunologic laboratory testing in the rheumatic diseases: anti-DNA antibody tests. *Arthritis & Rheumatism*.

[13] Fava M., Mallinckrodt C. H., Detke M. J., Watkin J. G., Wohlreich M. M. (2004). The effect of duloxetine on painful physical symptoms in depressed patients. *The Journal of Clinical Psychiatry*.

[14] Jennette J. C., Thomas D. B. (2001). Crescentic glomerulonephritis. *Nephrology Dialysis Transplantation*.

[15] Csernok E. (2003). Anti-neutrophil cytoplasmic antibodies and pathogenesis of small vessel vasculitides. *Autoimmunity Reviews*.

[16] Jennette J. C., Falk R. J., Bacon P. A. (2013). 2012 revised international Chapel Hill consensus conference nomenclature of vasculitides. *Arthritis & Rheumatism*.

[17] Jennette J. C., Wilkman A. S., Falk R. J. (1998). Diagnostic predictive value of ANCA serology. *Kidney International*.

[18] Chen M., Zhao M.-H., Zhang Y., Wang H. (2004). Antineutrophil autoantibodies and their target antigens in systemic lupus erythematosus. *Lupus*.

[19] M1 G., Morozzi G., Sebastiani G. D. (1998). Anti-neutrophil cytoplasmic antibodies in 566 European patients with systemic lupus erythematosus: prevalence, clinical associations and correlation with other autoantibodies. European Concerted Action on the Immunogenetics of SLE. *Clin Exp Rheumatol*.

[20] Manolova I., Dancheva M., Halacheva K. (2001). Antineutrophil cytoplasmic antibodies in patients with systemic lupus erythematosus: prevalence, antigen specificity, and clinical associations. *Rheumatology International*.

[21] Lee S. S., Lawton J. W., Chak W. (1991). Distinction between antinuclear antibody and P-ANCA. *Journal of Clinical Pathology*.

[22] Sen D., Isenberg D. A. (2003). Antineutrophil cytoplasmic autoantibodies in systemic lupus erythematosus. *Lupus*.

[23] Najafi C. C., Korbet S. M., Lewis E. J., Schwartz M. M., Reichlin M., Evans J. (2001). Significance of histologic patterns of glomerular injury upon long-term prognosis in severe lupus glomerulonephritis. *Kidney International*.

[24] Haas M., Eustace J. A. (2004). Immune complex deposits in ANCA-associated crescentic glomerulonephritis: a study of 126 cases. *Kidney International*.

